# Introduction to the *Toxins* Special Issue on Botulinum Toxins: New Uses in the Treatment of Diseases

**DOI:** 10.3390/toxins15090540

**Published:** 2023-09-01

**Authors:** Siro Luvisetto

**Affiliations:** National Research Council of Italy—CNR, Institute of Biochemistry and Cell Biology (IBBC), Via Ercole Ramarini 32, Monterotondo Scalo, 00015 Roma, Italy; siro.luvisetto@cnr.it

Studies on animals and humans have amply demonstrated the therapeutic efficacy of botulinum neurotoxins (BoNTs) in many pathologies. BoNTs act as blockers of cholinergic transmission at the neuromuscular junction (NMJ). This has led to the development of their clinical use in a variety of hypercholinergic disorders, such as dystonia, torticollis, blepharospasm and many others characterized by an abnormal release of acetylcholine at the NMJ. In the last decade, many studies have provided evidence of the efficacy of BoNTs in the treatment of pathologies where the expected therapeutic action is not limited only to NMJ, but is also attributable to the interaction of BoNTs with other structures or with neurotransmitters other than acetylcholine. Nowadays, the list of human pathologies in which treatment with BoNT produces beneficial effects is constantly increasing.

This Special Issue (SI), entitled: “Botulinum neurotoxins: new uses in the treatment of diseases”, is the third SI published by *Toxins* of which I am honored to be the Guest Editor. The previous SIs were dedicated to “Botulinum neurotoxins in the nervous system: future challenges for new indications” [1] and the “Effects of botulinum toxin on functional recovery after nervous system injuries”, whereby this SI, containing a collection of research on new therapy with BoNTs (mainly of the serotype A, BoNT/A), represents an update. This Editorial intends to introduce the 13 articles collected in this SI, which I strongly recommend reading in their original version.

The first contribution is a research article from Mueller et al. [2] reporting a human study on the effects of Xeomin^®^ (IncobotulinumtoxinA; a BoNT/A formulation specifically approved for the treatment of sialorrhea), concomitantly used with the radioligand actinium-225-PSMA in the therapy of prostate cancer metastases [2]. Cancer therapy with this radioligand compound is highly effective, as is its counterpart sialotoxic at the level of the parotid and submandibular glands, which regulate salivary secretions. Starting from the consideration that the injection into the salivary glands of Xeomin^®^ represents the elective therapy for chronic drooling, the authors had the excellent idea of using it to prevent excessive salivation during cancer therapy. The presented data demonstrated that an injection of a high dose, up to a total dose of 250 units of Xeomin^®^, into the salivary glands is well tolerated without causing severe systemic side effects. These findings paved the road to future trials that have included BoNT/A as a component for salivary gland protection in all of the radioligand cancer therapies, inducing permanent salivary gland dysfunction.

The next article, by Munoz-Lora et al. [3], presents new findings on the effect of BoNT/A in pain therapy, another booming BoNT/A therapeutic treatment. The authors analyzed the mechanism through which BoNT/A exerts antinociceptive effects on a rat model of temporomandibular joint (TMJ) rheumatoid arthritis, a chronic inflammatory pain model induced by repeated intra-articular injections of methylated bovine serum albumin in TMJ. The efficacy of two commercial pharmaceutical formulations of BoNT/A, namely Dysport^®^ (AbobotulinumtoxinA) and Botox^®^ (OnabotulinumtoxinA), was tested. Both formulations were able to reduce the pain-related behavior and the mechanical allodynia of the hypernociceptive rats. The antinociceptive effects of BoNT/A were correlated with the appearance of cleaved-SNAP-25 in the trigeminal nucleus caudalis, demonstrating an axonal transport of toxins to the central nociceptive sensory areas, together with reductions in c-Fos and GFAP upregulation, demonstrating a reduction in neuronal and glial activation.

Two other studies, by Yi et al. [4,5], provide anatomical information on the intramuscular neural arborization of the serratus anterior muscle, which is commonly injected with BoNTs for the treatment of myofascial pain syndrome, and various muscles surrounding the nasal region, which are frequently injected with BoNTs to increase effectiveness in removing wrinkles in the nose region. These two articles do not suggest a new BoNT treatment. However, they help to identify the exact anatomical distribution of specific muscles, such as the serratus anterior and the muscles of the nose region, which aids clinicians in electromyographic guidance of BoNT injections with the specific purpose of carefully targeting the correct muscles, avoiding adverse effects due to the probable spread of the toxin, especially after repeated treatments.

Another contribution of the present SI on the effects of BoNTs in the treatment of pain comes from a review article by Lippi et al. [6]. The authors reported a comparative analysis of 12 human studies published between 2006 and 2020, showing a significant effect of administering BoNT/A, various commercial preparations, or BoNT/B in patients suffering from neuropathic pain due to post-herpetic neuralgia, spinal cord injury, peripheral nerves, diabetic neuropathy, post-traumatic/post-operative neuropathies and carpal tunnel syndrome.

An innovative contribution regards the use of Botox^®^ (OnabotulinumtoxinA) in the field of dental occlusal treatment, specifically in a clinical application of the toxin to reconstruct the physiological homeostasis of the masticatory complex in patients affected by short-faced syndrome, presented by Li et al. [7]. The authors showed that by means of targeted injections of Botox^®^ into masseter muscle, to relax excessive powerful and thick muscle in short-faced patients, it was possible to restore the physiological homeostasis of the masticatory complex avoiding dental complications, such as temporomandibular disorders, bruxism, periodontitis, etc., often observed with other treatments.

A very exhaustive review, presented by Gazerani [8], is devoted to the effects of BoNTs in counteracting pruritus in a variety of conditions in which chronic itch is the main symptom. In the review, evidence of the beneficial effects of BoNTs is systematically analyzed using studies of both animal models or with healthy volunteers subjected to various forms of induced pruritus, or using off-label studies with humans subjected to chronic itchy conditions originating from different pathologies, whether dermatological or not. A mechanism of action of BoNTs is proposed, based on the blockade of the peripheral components of pruritus, i.e., the release of pruritic mediators and the activation of immune cells, blocking the vasomotor and autonomic components of pruritus.

A review article from Luvisetto [9] collected data on the interaction of BoNT/A with glial cells, both at the central (astrocytes, microglia and oligodendrocytes) and peripheral level (Schwann cells and satellite glial cells). An analysis of the data confirmed that BoNT/A can block the release of neuroactive substances not only from neuronal cells but also from glial cells. This effect is not limited to microglia and astrocytes but is also demonstrated in Schwan cells and oligodendrocytes, non-neuronal cells involved in the reconstruction of the myelin sheath damaged by traumatic injury. These results pave the way for an extraordinary application of BoNT/A in the treatment of spinal cord injuries, which will be the subject of further experimental research presented in this SI.

In another contribution, Wenninger et al. [10] analyzed the frequency of hemorrhagic side effects after BoNT/A injections, of different commercial preparations, for the treatment of benign essential blepharospasm and hemifacial spasm in patients taking antithrombotic drugs. The authors observed that, during the treatment of blepharospasm and hemifacial spasm with BoNT/A in patients undergoing concomitant treatment with antithrombotic drugs, the unwanted side effects, such as hematoma frequency, were not significantly different between patients treated with antithrombotic drugs or not. The main conclusion of this report was that the two treatments, both antithrombotic drugs and BoNT/A injection, can be safely performed together.

The next article is concerned with human studies combining the effect of BoNTs and the aid of electronic devices. Bertoncelli et al. [11] explained the use of a statistical machine learning algorithm, named BTX-PredictMed. The algorithm is based on a model that identifies clinical phenotypes for the prognosis of cerebral palsy in children that can benefit from an injection of BoNTs. The clinical features that the algorithm analyzes comprise neuromuscular scoliosis, equine foots, upper limbs and trunk muscle tone disorders, and the presence of other forms of spasticity, dystonia and dysplasia. The importance of this algorithm resides in the fact that it could be very helpful for professionals needing to make informed medical decisions.

In another study, Schulte-Baukloh et al. [12] reported an analysis of the exact onset of the action of Botox^®^ in patients affected by an overactive bladder. By means of a Diary Pod app, automated bladder diary equipment essentially constituting a measuring device connected through a mobile app to the remote dashboard of the clinical portal, the voiding frequency was accurately documented. A trend of reduction was observed 4 days after treatment with the toxin, with a significant change from the 5th day after toxin injection.

The extraordinary role of BoNT/A as an inducer of nerve regeneration in a mouse model of spinal cord injury was presented in the research article of Mastrorilli et al. [13]. In this study, a single dose of Xeomin^®^, injected directly into the spinal cord during the acute phase of traumatic injury, was able to induce complete regeneration and functional recovery, with the restoration of walking capacity, in paraplegic mice.

Finally, Beret et al. [14] show the effect of an intra-articular injection of Xeomin^®^ in a rat model of TMJ osteoarthritis induced via an intra-articular injection of monosodium iodoacetate. Behavioral, histological and radiographic analyses, with the latter performed using positron emission tomography imaging, showed reduced pain and decreased joint inflammation in the BoNT/A-treated animals.

In conclusion, the research and review articles included in this SI of *Toxins* contribute to advancing the state of the art on the novel therapeutic uses of BoNTs. Furthermore, many of the published studies focus on emerging or less-investigated applications of BoNTs in uncommon pathologies, thus providing the scientific community with new data supporting better knowledge of the contribution that can be made by BoNTs in improving human health.
